# Unravelling population genetic structure with mitochondrial DNA in a notional panmictic coastal crab species: sample size makes the difference

**DOI:** 10.1186/s12862-016-0720-2

**Published:** 2016-07-26

**Authors:** Sara Fratini, Lapo Ragionieri, Temim Deli, Alexandra Harrer, Ilaria A. M. Marino, Stefano Cannicci, Lorenzo Zane, Christoph D. Schubart

**Affiliations:** 1 Department of Biology, University of Florence, via Madonna del Piano 6, Sesto Fiorentino, Firenze 50019 Italy; 2 Institute for Zoology, Functional Peptidomics, University of Cologne, Cologne, Germany; 3 Laboratory of Genetics, Biodiversity and Enhancement of Bioresources (LR11ES41), University of Monastir, Higher Institute of Biotechnology of Monastir, Av. Tahar Hadded, B.P. 74, Monastir, 5000 Tunisia; 4 Zoology and Evolution, Faculty of Biology, University of Regensburg, D-93040 Regensburg, Germany; 5 Department of Biology, University of Padua, via U. Bassi 58/B, 35131 Padova, Italy; 6 The Swire Institute of Marine Science and School of Biological Sciences, The University of Hong Kong, Pokfulam Road, Hong Kong, Hong Kong SAR; 7 Consorzio Nazionale Interuniversitario per le Scienze del Mare, Piazzale Flaminio 9, 00196 Rome, Italy

**Keywords:** Phylogeography, Larval dispersal, Mediterranean Sea, Crustacea Brachyura, mtDNA CoxI

## Abstract

**Background:**

The extent of genetic structure of a species is determined by the amount of current gene flow and the impact of historical and demographic factors. Most marine invertebrates have planktonic larvae and consequently wide potential dispersal, so that genetic uniformity should be common. However, phylogeographic investigations reveal that panmixia is rare in the marine realm. Phylogeographic patterns commonly coincide with geographic transitions acting as barriers to gene flow. In the Mediterranean Sea and adjoining areas, the best known barriers are the Atlantic-Mediterranean transition, the Siculo-Tunisian Strait and the boundary between Aegean and Black seas. Here, we perform the so far broadest phylogeographic analysis of the crab *Pachygrapsus marmoratus*, common across the north-eastern Atlantic Ocean, Mediterranean and Black seas. Previous studies revealed no or weak genetic structuring at meso-geographic scale based on mtDNA, while genetic heterogeneity at local scale was recorded with microsatellites, even if without clear geographic patterns. Continuing the search for phylogeographic signal, we here enlarge the mtDNA dataset including 51 populations and covering most of the species’ distribution range.

**Results:**

This enlarged dataset provides new evidence of three genetically separable groups, corresponding to the Portuguese Atlantic Ocean, Mediterranean Sea plus Canary Islands, and Black Sea. Surprisingly, hierarchical AMOVA and Principal Coordinates Analysis agree that our Canary Islands population is closer to western Mediterranean populations than to mainland Portugal and Azores populations. Within the Mediterranean Sea, we record genetic homogeneity, suggesting that population connectivity is unaffected by the transition between the western and eastern Mediterranean. The Mediterranean metapopulation seems to have experienced a relatively recent expansion around 100,000 years ago.

**Conclusions:**

Our results suggest that the phylogeographic pattern of *P. marmoratus* is shaped by the geological history of Mediterranean and adjacent seas, restricted current gene flow among different marginal seas, and incomplete lineage sorting. However, they also caution from exclusively testing well-known biogeographic barriers, thereby neglecting other possible phylogeographic patterns. Mostly, this study provides evidence that a geographically exhaustive dataset is necessary to detect shallow phylogeographic structure within widespread marine species with larval dispersal, questioning all studies where species have been categorized as panmictic based on numerically and geographically limited datasets.

**Electronic supplementary material:**

The online version of this article (doi:10.1186/s12862-016-0720-2) contains supplementary material, which is available to authorized users.

## Background

Terrestrial and marine biogeographic regions all over the world are defined based on the distribution of extant species. Boundaries separating adjacent regions may derive from historical breaks or from present-day environmental differences and may contribute to giving rise to genetic discontinuities within species and to allopatric speciation events.

The Mediterranean Sea has a long and complex geological history that has contributed to its high biodiversity and high proportion of endemisms [1]. This epeiric sea is a remnant of the Tethys Sea, and since the Miocene it experienced periods of desiccations (the Messinian Salinity Crisis around 5.5 Mya as well as during various Quaternary glaciations) and subsequent flooding from the Atlantic Ocean. The current biota is therefore mainly linked to colonization from the Atlantic, after the opening of the Strait of Gibraltar in the early Pliocene [1].

**Fig. 1:**
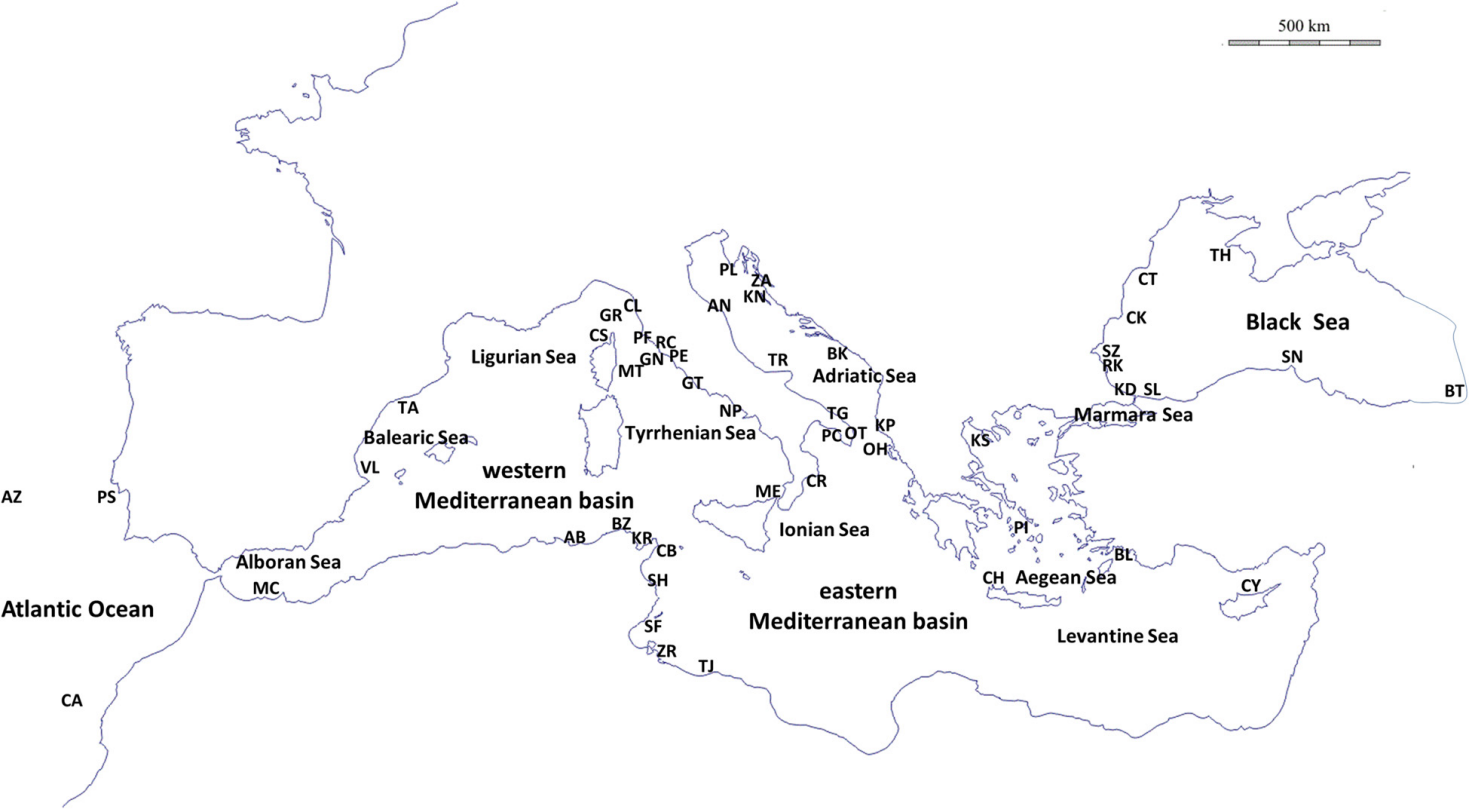
Atlantic, Mediterranean and Black Sea localities analysed for *Pachygrapsus marmoratus*. Details on sampling sites are reported in Table 1. Basins and sub-basins of the Mediterranean Sea are indicated

Based on range distributions, community composition of marine fauna and differences in salinity and winter surface temperatures, the Mediterranean Sea can be subdivided in two main basins, a western and an eastern one, separated by the Strait of Sicily [2]. Moreover, both basins can be subdivided into sub-basins (Fig. 1): the western basin includes the Alboran Sea, in strict connection with the Atlantic Ocean, Balearic Sea, Ligurian Sea and Tyrrhenian Sea (Fig. 1); while the eastern basin includes the Ionian Sea, the Aegean Sea, communicating with the Black Sea through the Marmara Sea, and the Levantine Sea, now in direct connection with the Red Sea (Fig. 1). The Adriatic Sea is separated from the rest of the eastern basin by the Strait of Otranto.

Boundaries among basins and sub-basins can act as barriers to dispersal of marine organisms at different orders of magnitude, shaping intra- and inter-specific marine diversity (reviewed in [3]). Evident phylogenetic and phylogeographic breaks are those associated with two main geographical transitions: the Atlantic-Mediterranean transition at Strait of Gibraltar or the Almería-Oran Front, and the transition separating the western and eastern Mediterranean basins at the Strait of Sicily (reviewed in [3]). For example, Zane et al. [4] and Papetti et al. [5] recorded the occurrence of separated evolutionary units of the euphausiid *Meganyctiphanes norvegica* corresponding to the north-eastern Atlantic, the western Atlantic, the Alboran Sea and the Mediterranean Sea. The swordfish *Xiphias gladius* is another example of genetic isolation among Atlantic and Mediterranean populations [6] and genetic distinctiveness of eastern and western Mediterranean populations [7]. A similar pattern is also reported for the European spiny lobster *Palinurus elephas* notwithstanding its long larval phase, with the existence of separated gene pools in the Atlantic Ocean, the western and eastern Mediterranean Sea, respectively [8–10].

Another relevant phylogeographic transition is the boundary between the eastern Mediterranean basin and the Black Sea through the Marmara Sea. The Black Sea is a relatively small semi-enclosed body of water differentiated from the adjacent Aegean Sea in terms of its chemo-physical conditions and biodiversity [11]. Currently, this area is connected by a two-way water exchange, but this connection is relatively recent, since during the last glacial maximum, 20,000 years ago, the global sea level was around 120 m below its present one. At that time, the Black Sea was totally isolated from the Mediterranean Sea and was successively diluted with fresh water [12]. In recent years, genetic studies have reported striking examples of separated lineages due to this phylogeographic break. The Black Sea bottlenose dolphins *Tursiops truncatus*, for example, are genetically and morphologically differentiated from Mediterranean and Atlantic conspecifics [13], while the porpoise *Phocoena phocoena* is represented by an endemic subspecies, *P. phocoena relicta,* in the Black Sea [14]. Copepods of the genus *Calanus* constitute another well-studied example of marine invertebrates whose Black Sea populations are genetically separated from those of the Atlantic, western Mediterranean, Adriatic and Aegean populations [15].

Most of these examples of intraspecific differentiation refer to pelagic species, while few ones report about the influence of seal level changes on benthic coastal species. Thus, the aim of this study is to investigate intraspecific differentiation in one of the most common, widely distributed and best known invertebrate species of the Mediterranean Sea, the marbled crab *Pachygrapsus marmoratus* (Fabricius, 1787) (Decapoda; Brachyura; Thoracotremata; Grapsidae). This species occupies the upper and middle shore levels of the rocky coasts of the Mediterranean Sea, Black Sea and north-eastern Atlantic Ocean, from Brittany to Morocco, including the Canary Islands, the Azores, and Madeira [16, 17]. Notwithstanding its huge distribution range, the species’ suitable habitats over the entire distribution range are locally separated by extensive stretches of sandy beaches. Adults are relatively sedentary [18], and thus connectivity among populations is maintained by the larval stages, developing for about four weeks in the water column [19] before the megalopa re-colonises the coastal habitat. Sequence variation of the mitochondrial DNA (mtDNA) so far indicated rather high levels of genetic exchange in the species at meso-geographic scales, with a weak separation among Atlantic and Mediterranean populations and lack of differentiation within the Mediterranean Sea [20, 21]. Conversely, some genetic heterogeneity at local scales emerged from previous studies investigating microsatellite polymorphisms [22–24]. Overall, these evidences suggest weak departure from panmixia, as initially supposed for this species and call for investigation of the likely influence of different geographic breaks across its distribution range.

In this study, we gathered a very large dataset to investigate regional population genetic structure and phylogeographic pattern of the marbled crab *P. marmoratus* covering the Atlantic Ocean, the various basins and sub-basins of the Mediterranean Sea and the Black Sea (Fig. 1). With respect to the earlier genetic studies [20, 21], new populations from the Adriatic, northern Aegean and Black Sea were included, allowing an extensive investigation of phylogeographic patterns over a large part of the distributional range of *P. marmoratus*. A total of 587 samples from 51 populations were screened for sequence variation at the mitochondrial cytochrome oxidase subunit 1 (mtDNA CoxI) with the twofold aim of: 1) depicting the effect of currently recognised biogeographic breaks on the geographical distribution of intraspecific genetic variation; and 2) uncovering how historical events may have contributed to shape patterns of intra- and inter-population genetic diversities.

## Methods

### Study area

A total of 587 samples of *Pachygrapsus marmoratus* were included in the present phylogeographic study, of which 246 were specifically analysed de novo and the remaining 341 had been previously published: 238 sequences from western Mediterranean and Atlantic by Fratini et al. [20], 98 sequences from North Africa and Turkey by Deli et al. [21] and five sequences from the Azores by Matzen da Silva et al. [25]. Specimens were collected from 51 localities of the Mediterranean Sea, Black Sea and Atlantic Ocean, covering most of the distribution range of the species (Table 1 and Fig. 1). For most samples, a chela or a pereiopod was preserved in absolute ethanol, and the animals released. Details on the sampled localities and the number of individuals analysed per population are reported in Fig. 1 and Table 1.

**Table 1:** *Pachygrapsus marmoratus* populations sampled from the Atlantic Ocean, Mediterranean Sea, and Black Sea

#	Area	Nation	Population	Cod.	GPS		N
1	Atlantic Ocean	Portugal	Sesimbra	PS	38° 26.37′ N	09° 06.92′ W	18
2		Spain	Canary Isl: Fuerteventura	CA	28° 03.14′ N	14° 21.85′ W	18
3		Portugal	Azores Isl: Terciera	AZ	38° 48.10′ N	27° 15.15′ W	17
4	Mediterranean Sea: Alboran Sea	Morocco	Cala Iris	MC	35° 09.10′ N	4° 21.59′ W	15
5	Mediterranean Sea: Balearic Sea	Spain	Tarragona	TA	41° 06.40′ N	01° 14.86′ E	20
6		Spain	Valencia	VL	39° 26.80′ N	0° 19.19′ W	20
7	Mediterranean Sea: Ligurian Sea	France	Corsica: St. Florent	CS	42° 40.82′ N	09° 17.80′ E	15
8	Mediterranean Sea: Tyrrhenian Sea and opposite North African coastline	Italy	Calafuria	CL	43° 28.32′ N	10° 20.01′ E	15
9		Italy	Porto di Follonica	PF	42° 53.26′ N	10° 47.2′ E	5
10		Italy	Porto Ercole	PE	42° 23.65′ N	11° 12.33′ E	15
11		Italy	Giglio Isl.	GN	42° 21.54′ N	10° 55.27′ E	11
12		Italy	Rocchette	RC	42° 46.58′ N	10° 47.59′ E	14
13		Italy	Montecristo Isl.	MT	42° 20.63′ N	10° 19.28′ E	15
14		Italy	Gorgona Isl.	GR	43° 25.87′ N	09° 54.40′ E	9
15		Italy	Gaeta	GT	41° 12.38′ N	13° 34.26′ E	15
16		Italy	Fusaro	NP	40° 48.80′ N	14° 02.44′ E	15
17		Algeria	Annaba	AB	36° 54.00′ N	07° 45.14′E	10
18		Tunisia	Bizerte	BZ	37° 16.13′ N	09° 52.01′ E	8
19		Tunisia	Korbos	KR	36° 49.10′ N	10° 34.01′ E	9
20	Mediterranean Sea: Ionian Sea and opposite North African coastline	Italy	Crotone	CR	39° 05.60′ N	17° 07.68′ E	16
21		Italy	Messina	ME	38° 11.46′ N	15° 33.49′ E	16
22		Italy	Porto Cesareo	PC	40° 11.72′ N	17° 55.08′ E	7
23		Tunisia	Cap Bon	CB	36° 51.77′ N	11° 05.19′ E	12
24		Tunisia	Sahel	SH	36° 10.46′ N	10° 48.93′ E	11
25		Tunisia	Sfax	SF	34° 44.14′ N	10° 45.00′ E	9
26		Tunisia	Zarzis	ZR	33° 30.34′ N	11° 06.21′ E	11
27		Lybia	Tajura	TJ	32° 52.00′ N	13° 20.43′ E	10
28	Mediterranean Sea: Adriatic Sea	Greece	Othonoi	OH	39° 14.18′ N	20° 28.72′ E	7
29		Italy	Ancona	AN	43° 36.62′ N	13° 29.10′ E	7
30		Italy	Otranto	OT	40° 06.55′ N	18° 31.15′ E	7
31		Italy	Torre Guaceto	TG	40° 43.00′ N	17° 48.00′ E	7
32		Italy	Tremiti: Capraia Is.	TR	42° 08.32′ N	15° 31.44′ E	7
33		Albania	Karaburun Peninsula	KP	40° 23.57′ N	19° 19.50′ E	7
34		Croatia	Kornati	KN	43° 47.54′ N	15°16.89′ E	7
35		Croatia	Pula	PL	44° 52.12′ N	13° 50.27′ E	18
36		Croatia	Zadar	ZA	44° 07.32′ N	15° 13.45′ E	11
37		Montenegro	Boka Kotorska Bay	BK	42° 23.25′ N	18° 34.18′ E	7
38	Mediterranean Sea: Aegean Sea and Levantine Sea	Greece	Crete: Iraklion harbour	CH	35° 20.41 N	25° 08.15′ E	14
39		Greece	Paros Naoussa harbour	PI	37° 07.52′ N	25° 14.24′ E	7
40		Greece	Chalkidiki: Kassandra: Possidi	KS	39° 57.62′ N	23° 23′52 E	8
41		Turkey	Lycia: Beldibi	BL	36° 52.36′ N	28° 15.97′ E	18
42		Cyprus	Girne	CY	35° 20.56′ N	33° 18.07′ E	22
43	Black Sea	Turkey	Sile	SL	41° 10.99′ N	29° 36.74′ E	7
44		Turkey	Sinop	SN	42° 00.96′ N	35° 10.96′ E	7
45		Bulgaria	Sozopol	SZ	42° 25.02′ N	27° 41.46′ E	21
46		Bulgaria	Cape Kaliakra	CK	43° 24.71′ N	28° 21.00′ E	7
47		Bulgaria	Ropotamo-Kiten	RK	42° 11.71′ N	27° 50.16′ E	7
48		Georgia	Batumi	BT	41° 41.28′ N	41° 42.08′ E	7
49		Ukraine	Karadag	KD	44° 54.37′ N	35° 15.33′ E	7
50		Ukraine	Tarhankut	TH	45° 20.03′ N	32° 33.09′ E	7
51		Romania	Costinesti	CT	43° 55.53′ N	28° 38.44′ E	7
	Overall	/	/	O	/	/	587
	Atlantic Ocean	/	/	A	/	/	53
	Mediterranean Sea	/	/	M	/	/	457
	Black Sea	/	/	B	/	/	77

For each site indicated the provenience basin/sub-basin; the nation; the sampling location; the abbreviation code for populations; the geographical coordinates; the number of analysed specimens are reported.

### Genetic analysis

Total genomic DNA extraction was performed from muscle tissue using the Puregene Kit (Gentra Systems) or the Salting Out extraction method [26]. DNAs were resuspended in sterile distilled water and stored at 4 °C for routine use, or at −20 ° C for long-term storage.

Selective amplification of 658 basepairs of the mtDNA CoxI was performed as reported in Fratini et al. [20], using the primers COL6b (5′-acaaatcataaagatatygg-3′) [27] and HCO2198 (5′-taaacttcagggtgaccaaaaaatca-3′) [28]. Amplicons were visualized on an 1 % agarose gel, purified by precipitation with Sure Clean (Bioline), resuspended in water, and then sequenced with the ABI BigDye terminator mix followed by electrophoresis in an ABI Prism 310 Genetic Analyzer (Applied Biosystem, Foster City, USA) at the University of Florence (Italy), University of Regensburg (Germany) and at the BMR-Genomics (http://www.bmr-genomics.it). Unpublished haplotypes were submitted to molecular databases (accession numbers KX529672-KX529696). The sequences were corrected manually with the program CHROMAS version 1.55 (Technelysium Pty Ltd, Queensville, Australia) and aligned by eye with BioEdit ver. 7.2.5 [29].

The nucleotide composition as well as the number and type of mutations were calculated with MEGA6 [30]. The number of haplotypes, the unbiased haplotype diversity corrected for sample size (h, calculating using the Nei’s [31] formula h = (1 ‐ Σx_i_^2^)n/n ‐ 1, where x_i_ is the frequency of a haplotype and n is the sample size), and the nucleotide diversity (π_π_: the mean number of differences between all pairs of haplotypes in a population, expressed as percentage) [31] were calculated for each population, the overall population and geographic groups of populations, using the software ARLEQUIN ver. 3.5.2.2 [32].

A minimum spanning network was built with NETWORK version 4.5.0.1 (Shareware Phylogenetic Network Software; http://www.fluxus-engineering.com/sharenet.htm) to assess the intra-specific evolutionary relationships among the haplotypes of *P. marmoratus*. Genetic differentiation among populations was estimated by one-way AMOVA [33], as implemented in ARLEQUIN. Fixation indices (*Ф*) [34] were computed using genetic distances (Tajima and Nei model, suggested for unequal nucleotide frequencies [35]). Additional two-way AMOVAs were performed for testing specific biogeographic hypotheses (see Results). Significance levels of pairwise *Ф*st values, under the null hypothesis of no differentiation, were computed by permutation tests from 10,000 random permutations of haplotypes between populations and, when appropriate, populations between groups. When needed, multitest corrections were performed following the B-H method [36] using the program SGoF+ ([37]; http://webs.uvigo.es/acraaj/SGoF.htm).

To visualize the occurrence of genetic structure and setting our biogeographic hypotheses, we performed a Principal Coordinates Analysis (PCoA), based on the genetic distances among all pairwise combinations of populations (expressed as *Ф*st pairwise values). The analysis was run using the covariance-standardized method as implemented in GenAlex 6.5 [38].

To infer the demographic history of our populations, we applied three neutrality tests to each population, the overall population, and geographic groups of populations: the Tajima’s D [39] and the R2 test [40] that use information on the mutation frequency and are appropriate for distinguishing population growth from constant size population; and the Fu’s Fs test which is based on information from the haplotype distribution and is more sensitive to the presence of singletons in a sample [41, 42]. The Tajima’s D and Fu’s Fs parameters (both expected to be equal to zero under the hypothesis of selective neutrality and population equilibrium) were assessed as implemented in ARLEQUIN, and their significance levels were calculated by generating 1,000 random samples. Significant negative D and Fs values can be interpreted as signatures of population expansion. The R2 test was calculated using DnaSP ver. 5.10 [43] and its significance level was estimated based on 1,000 simulated re-sampling replicates.

Since departures from neutrality are often due to changes in effective population size, we also applied Bayesian Skyline plots (BSP) [44] to fit demographic models for *P. marmoratus*, as implemented in BEAST ver. 1.8.3 [45]. BSP plots were generated for the genetically homogeneous geographic groups inferred by AMOVA (see Results section). For the analysis we used a HKY + I model of mutation, the closest model available in BEAST to the model TrN + I that was suggested by jMODELTEST version 2.1.10 [46, 47], and a strict molecular clock. Due to the low sample size and the corresponding low number of haplotypes, the Markov-Chain Monte Carlo (MCMC) simulations did not reach convergence for Atlantic populations and results are reported only for the Black and the Mediterranean seas. For the Black Sea, two MCMC runs of 100,000,000 iterations, sampling every 100,000 steps, were performed. In the case of the Mediterranean Sea, the two MCMC were run for 1,000,000,000 iterations, sampling every 500,000 steps. Both for the Black Sea and the Mediterranean runs, the first 10 % iterations were discarded as burn-in and LogCombiner [45] was used to combine the replicates. TRACER 1.6 [48] was used to check convergence by measuring Effective Sample Sizes (ESS) of all parameters (ESS > 200 for each group) and to calculate the mean value, the upper and lower bounds of the 95 % highest posterior density interval of effective population sizes, and to draw skyline plots. Estimation of time since expansion event was inferred from converting mutations units in estimates of years using a CoxI mutation rate of 1.66 % per million years [49].

## Results

Our mtDNA CoxI alignment was cropped to a length of 596 basepairs. It has an A-T rich nucleotide composition (C = 21.0 %, T = 35.9 %, A = 25.1 % and G = 18.0 %), as commonly found in the mitochondrial DNA of arthropods [50]. The dataset included 74 haplotypes, of which 25 are new to science (haplotypes 50–74) (Additional file 1). Haplotypes 1–33 (accession numbers JF930650-82) were already reported in Fratini et al. [20], and haplotypes 34–49 (accession numbers KX549320-KX549335) in Deli et al. [21]. Five sequences from Azores were downloaded from GenBank (accession numbers JQ306088-92; [25]) and correspond to our haplotype 2. Among the 74 haplotypes, we recorded a total of 59 variables sites, of which 26 are parsimony-informative and 33 singletons (Additional file 1). Most of the mutations are in third triplet positions and only seven are not silent (Additional file 1).

**Fig. 2:**
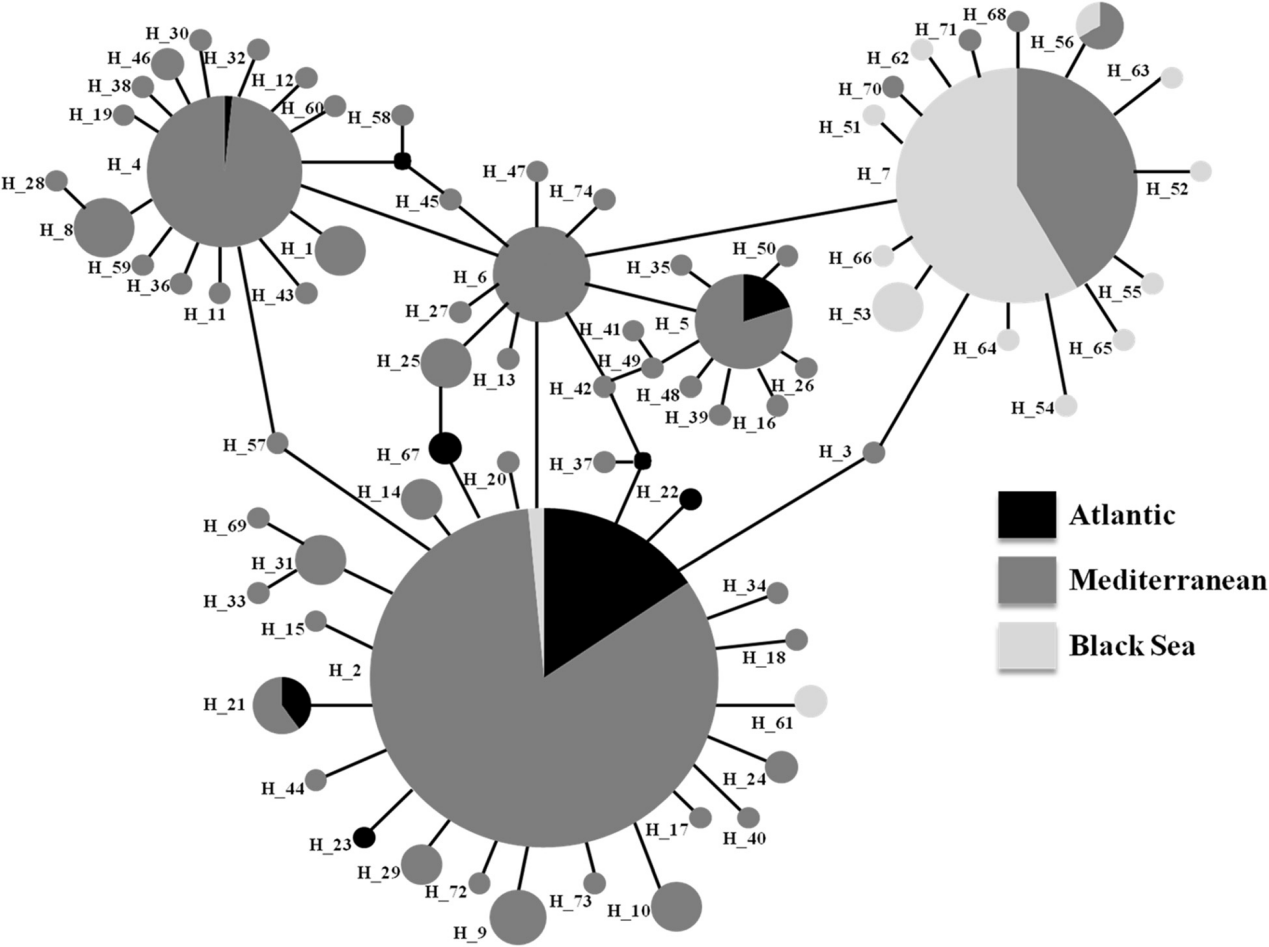
Minimum spanning network showing the relationships among the recorded haplotypes of *Pachygrapsus marmoratus*. Each line represents one mutational step. Circles representing haplotypes are scaled to their frequencies. H6 represents the ancestral haplotype

Most haplotypes differ from each other by single or very few mutations (Fig. 2; Additional file 1). The network is well resolved with three main haplotypes; about 73 % of all individuals carry one of these common haplotypes. Haplotype 2 is the most common one and is present in 271 individuals (corresponding to 46.2 %): 225 out of 457 from the Mediterranean Sea (more than 49 %), 42 out of 53 from the Atlantic Ocean (around 80 %), but only 3 of 77 from the Black Sea (around 4 %). Haplotype 7 is present in 96 individuals and is restricted to the Mediterranean and Black seas, with about 8.5 % (40 individuals) and 73 % (56 individuals) of their sampled specimens, respectively. Haplotype 4 is present in a total of 62 (10.6 %) individuals, 61 collected in the Mediterranean Sea (12.5 % of its individuals) and one in the Atlantic Ocean. Haplotype 6 seems to be the ancestral haplotype showing the highest number of connections. In addition, 54 haplotypes (i.e. about 73 %) are singletons, i.e. present only in one individual. Ten of the fourteen haplotypes found in the Black Sea are restricted to this geographic area, and most of them seem to originate from haplotype 4. The association between haplotypes and geography is less evident for the Atlantic Ocean, even if three out of seven haplotypes found in this area are endemic. Both Atlantic and Black Sea populations seem to originate from Mediterranean ones.

The average haplotype and nucleotide diversities are 0.75 ± 0.02 and 0.27 ± 0.1, respectively (Table 2). All populations are characterised by low nucleotide diversity indices (<0.5 %), while haplotype diversity values per population vary from a minimum of 0.0 ± 0.0 to a maximum of 0.97 ± 0.06 (Table 2). Calculating genetic diversity indices for the three main geographic groups (i.e. Atlantic Ocean, Mediterranean and Black seas), it becomes evident that the Mediterranean Sea has on average higher haplotype and nucleotide diversities in comparison to the Atlantic Ocean and Black Sea (Table 2).

**Table 2:** Genetic diversity and historical demographic results for populations of *Pachygrapsus marmoratus* from the Atlantic Ocean, Mediterranean Sea and Black Sea

#	Area	Cod.	N	Nhap	h	π (%)	T-D	F-Fs	R2
1	Atlantic Ocean	PS	18	3	0.22 ± 0.12	0.01 ± 0.01	**−1.71**	−1.02	0.17
2		CA	18	6	0.62 ± 0.12	0.18 ± 0.14	−1.21	**−2.14**	**0.09**
3		AZ	17	2	0.22 ± 0.12	0.04 ± 0.05	−0.49	0.03	**0.16**
4	Mediterranean Sea: Alboran Sea	MC	15	6	0.57 ± 0.15	0.15 ± 0.12	−1.45	−3.23	**0.09**
5	Mediterranean Sea: Balearic Sea	TA	20	9	0.82 ± 0.07	0.28 ± 0.19	−0.89	**−4.14**	**0.09**
6		VL	20	5	0.44 ± 0.13	0.18 ± 0.14	−1.44	−0.85	0.1
7	Mediterranean Sea: Ligurian Sea	CS	15	7	0.72 ± 0.12	0.25 ± 0.18	−0.72	**−3.38**	0.11
8	Mediterranean Sea: Tyrrhenian Sea and opposite North African coastline	CL	15	5	0.64 ± 0.13	0.24 ± 0.17	−0.28	−0.67	0.13
9		PF	5	1	0.0 ± 0.0	0.0 ± 0.0	0.0	0.0	0.0
10		PE	15	3	0.45 ± 0.13	0.13 ± 0.11	0.63	0.36	0.19
11		GN	11	5	0.78 ± 0.09	0.24 ± 0.0	0.14	−1.2	0.16
12		RC	14	7	0.81 ± 0.09	0.30 ± 0.21	−0.14	**−2.38**	0.13
13		MT	15	9	0.85 ± 0.09	0.32 ± 0.22	−1.17	**−6.05**	**0.08**
14		GR	9	7	0.92 ± 0.09	0.39 ± 0.26	−1.03	**−4.71**	**0.10**
15		GT	15	67	0.77 ± 0.10	0.31 ± 0.21	−1.22	**−2.1**	**0.09**
16		NP	15	5	0.63 ± 0.12	0.23 ± 0.17	−0.36	−0.74	0.13
17		AB	10	6	0.84 ± 0.10	0.37 ± 0.25	−0.43	−1.56	0.14
18		BZ	8	5	0.79 ± 0.15	0.32 ± 0.23	−1.36	−1.23	**0.12**
19		KR	9	8	0.97 ± 0.06	0.47 ± 0.31	−0.74	**−4.62**	**0.12**
20	Mediterranean Sea: Ionian Sea and opposite North African coastline	CR	16	5	0.53 ± 0.14	0.18 ± 0.14	−0.89	−1.18	**0.11**
21		ME	16	7	0.82 ± 0.07	0.29 ± 0.20	−0.66	−2.19	0.12
22		PC	7	3	0.67 ± 0.17	0.24 ± 0.19	0.75	0.67	0.22
23		CB	12	6	0.76 ± 0.12	0.27 ± 0.20	−0.67	−1.87	**0.12**
24		SH	11	4	0.60 ± 0.15	0.17 ± 0.14	−0.93	−0.7	**0.13**
25		SF	9	6	0.89 ± 0.09	0.29 ± 0.21	−0.27	**−2.58**	0.15
26		ZR	11	7	0.82 ± 0.12	0.36 ± 0.24	−1.27	**−2.65**	**0.09**
27		TJ	10	5	0.67 ± 0.16	0.25 ± 0.18	−1.28	−1.32	**0.12**
28	Mediterranean Sea: Adriatic Sea	OH	7	3	0.77 ± 0.11	0.26 ± 0.20	1.11	**0.79**	0.25
29		AN	7	6	0.95 ± 0.9	0.41 ± 0.29	−0.69	−2.7	**0.15**
30		OT	7	3	0.67 ± 0.16	0.27 ± 0.21	−0.04	0.9	0.22
31		TG	7	6	0.95 ± 0.09	0.30 ± 0.23	−0.56	**−3.55**	**0.14**
32		TR	7	3	0.71 ± 0.13	0.20 ± 0.19	0.75	0.67	0.22
33		KP	7	3	0.67 ± 0.16	0.29 ± 0.22	0.24	1.01	0.23
34		KN	7	4	0.86 ± 0.10	0.21 ± 0.17	0.05	−1.06	0.20
35		PL	18	6	0.81 ± 0.06	0.23 ± 0.16	−0.2	−1.54	0.13
36		ZA	11	6	0.84 ± 0.09	0.25 ± 0.18	0.34	**−2.36**	0.18
37		BK	7	5	0.86 ± 0.14	0.37 ± 0.26	−0.54	−1.35	**0.15**
38	Mediterranean Sea: Aegean Sea and Levantine Sea	CH	14	3	0.58 ± 0.09	0.17 ± 0.14	1.7	0.9	0.26
39		PI	7	5	0.86 ± 0.14	0.29 ± 0.22	−0.79	**−1.89**	**0.11**
40		KS	8	5	0.76 ± 0.15	0.26 ± 0.20	−0.92	**−1.75**	**0.11**
41		BL	18	6	0.68 ± 0.11	0.19 ± 0.15	−0.66	−2.02	0.11
42		CY	22	9	0.74 ± 0.09	0.25 ± 0.18	−1.04	−4.27	**0.09**
43	Black Sea	SL	7	3	0.67 ± 0.16	0.13 ± 0.11	−0.27	1.22	0.21
44		SN	7	1	0.0 ± 0.0	0.0 ± 0.0	0.0	0.0	0.0
45		SZ	21	7	0.56 ± 0.13	0.17 ± 0.13	**−2.0**	**−3.33**	**0.08**
46		CK	7	4	0.81 ± 0.13	0.29 ± 0.22	0.24	−0.43	0.20
47		RK	7	4	0.71 ± 0.18	0.19 ± 0.16	−1.43	−1.22	0.18
48		BT	7	2	0.29 ± 0.20	0.14 ± 0.13	−1.36	1.51	0.35
49		KD	7	2	0.29 ± 0.20	0.05 ± 0.06	−1.01	−0.09	0.35
50		TH	7	1	0.0 ± 0.0	0.0 ± 0.0	0.0	0.0	0.0
51		CT	7	3	0.52 ± 0.21	0.10 ± 0.10	−1.24	−0.92	0.23
	**Overall**	O	587	74	0.75 ± 0.00	0.27 ± 0.18	**−2.26**	**−27.11**	**0.01**
	**Atlantic Ocean**	A	53	7	0.37 ± 0.08	0.10 ± 0.08	**−1.63**	**−3.99**	**0.16**
	**Mediterranean Sea**	M	457	60	0.73 ± 0.02	0.26 ± 0.17	**−2.23**	**−27.28**	**0.07**
	**Black Sea**	B	77	14	0.45 ± 0.07	0.14 ± 0.11	**−2.14**	**−12.48**	**0.03**

Population and area codes correspond to those reported in Table 1. Values reported are: number of individuals analysed (N); number of haplotypes (Nhap); haplotype (h) and nucleotide (π) diversity (the latter expressed in percentage); T-D, Tajima D test; F-Fs, Fu’s Fs test; R2, Ramos-Onsins and Rozas R2 test. Significant values (*P* < 0.05) are in bold

The one-way AMOVA test, based on Tajima and Nei molecular distances, indicates the existence of genetic differentiation among populations (*Ф*st = 0.18, *P* < 0.001). After correcting for multiple tests with the B-H method [36], 394 out of 1275 tests result to be statistically significant based on *Ф*st pairwise values. The Black Sea populations are genetically separated from all the Atlantic populations with very high *Ф*st values (from a maximum of 0.92 recorded for AZ vs TH to a minimum of 0.38 for CK vs CA) and from most of the Mediterranean populations (Additional file 2). Many significant pairwise comparisons also regard two Atlantic populations, PS and AZ, with respect to the Mediterranean populations (Additional file 2); while only few *Ф*st pairwise values involving the comparison between two Mediterranean populations are statistically significant (Additional file 2). The populations from Alboran Sea and from Canary Islands do not show any significant pairwise *Ф*st values in comparison to Atlantic populations from Portugal and Azores, and at the same time they are genetically not differentiated from most of Mediterranean populations (Additional file 2).

**Fig. 3:**
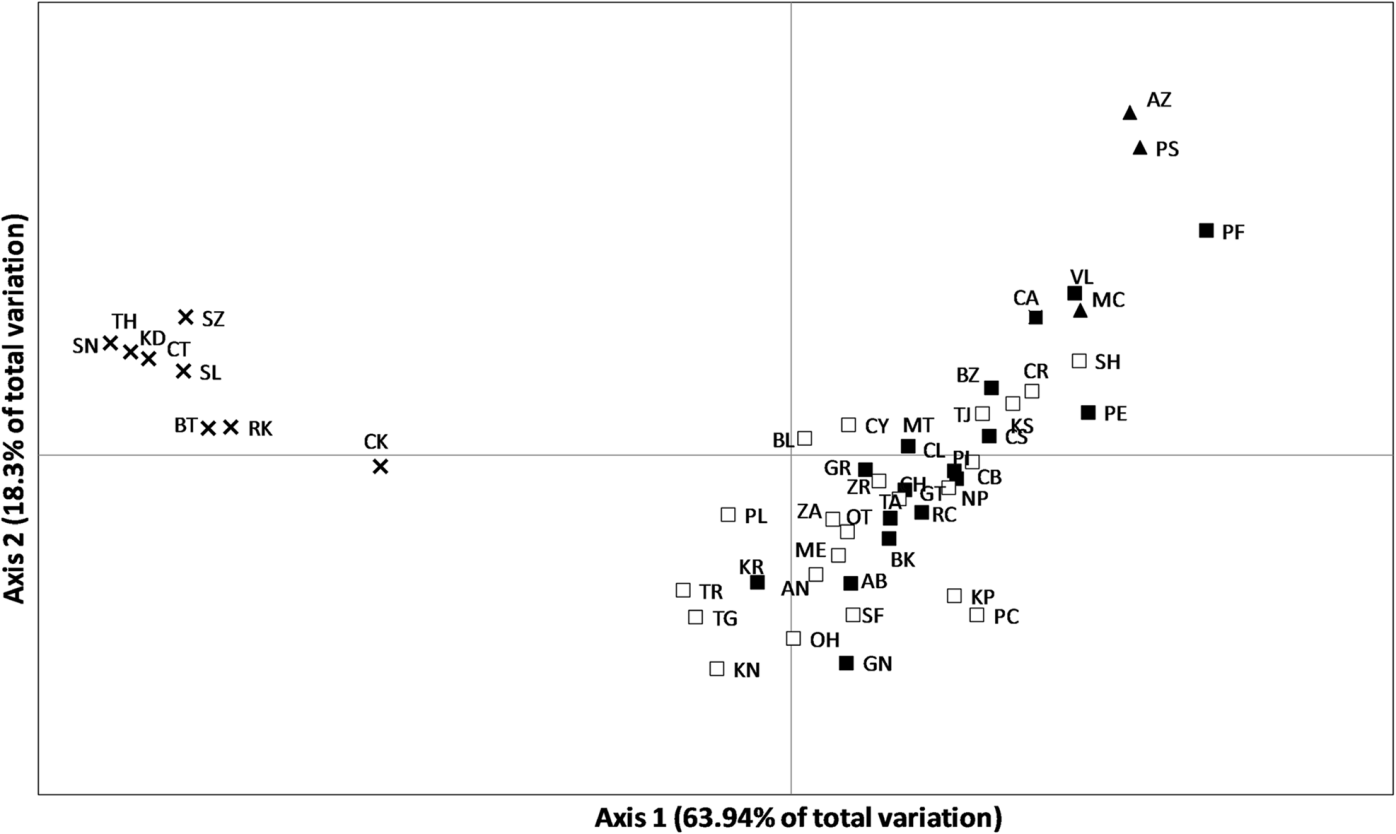
Principal Coordinates Analysis (PCoA) plot based on genetic distances (expressed as *Φ*st pairwise values) among Atlantic Ocean (solid triangles), western (solid squares) and eastern (empty squares) Mediterranean Sea, and Black Sea (crosses) populations of *Pachygrapsus marmoratus*. Each symbol is a population, acronyms are reported in Table 1

The PCoA analysis, with axis 1 and axis 2 explaining 63.94 % and 18.3 % of the distribution, respectively, confirm a clear-cut genetic separation of Black Sea populations from Mediterranean and Atlantic ones (Fig. 3). The existence of a separate lineage in the Black Sea is corroborated by the fact that the samples collected in the three geographically closest Mediterranean sites (KS, PI and BL) are not the closest ones in the PCoA ordination, as expected in case of isolation by distance. With regard to the Atlantic samples, only the two northern populations, PS and AZ, cluster together, while the population from Canary Island, CA, cluster within the Mediterranean group and appears to be closely related to MC and VL (i.e. the populations from Alboran and Balearic Sea) (Fig. 3). The PCoA ordination does not reveal any clear subdivision among the Mediterranean populations, confirming the lack of genetic separation between western and eastern basins (Fig. 3).

Based on the *Ф*st pairwise comparison values, on the PCoA plot and on the network, we tested some alternative biogeographic hypotheses by applying 2-way AMOVAs (Table 3). First, we grouped populations into three geographic groups corresponding to Atlantic Ocean (PS + AZ + CA), Mediterranean Sea, and Black Sea: the analysis recorded a *Ф*ct equal to 0.34 (*P* < 0.001). Alternatively, we included the Alboran population within the Atlantic group, for testing the hypothesis that the Atlantic-Mediterranean transition could be located at the Almería-Oran Front in line with the break reported for other species by Patarnello et al. [3]: in this case, the recorded *Ф*ct were slightly lower, being equal to 0.32 (*P* < 0.001). When the Atlantic population from Canary Islands (CA) was grouped with the Mediterranean populations, in line with the PCoA results, the *Ф*ct value increased to 0.36 (*P* < 0.001). Finally, a separation among the main Mediterranean basins was not underlined from a further 2-way AMOVA, splitting the Mediterranean group into three subgroups (i.e. five groups; western Mediterranean + CA/Adriatic Sea/eastern Mediterranean/Atlantic/Black Sea), as indicated from the lower *Ф*ct value respect to those associated to the previous biogeographic hypotheses (*Ф*ct = 0.21, *P* < 0.001).

**Table 3:** Analysis of molecular variance testing for partitioning of *Pachygrapsus marmoratus* genetic variation under alternative biogeographic hypotheses

Hypothesis	Source of variation	df	F-statistics	*P*
1) three groups, geography				
- Atlantic (PS + AZ + CA)	Among groups	2	*Φ*ct = 0.34	**<0.001**
- Mediterranean Sea (including Alboran Sea, MC)	Among pops/within groups	47	*Φ*sc = 0.02	0.067
- Black Sea	Among populations	530	*Φ*st = 0.35	**<0.001**
2) three groups, Orano-Almeria front				
- Atlantic and Alboran Sea (PS + AZ + CA + MC)	Among groups	2	*Φ*ct = 0.32	**<0.001**
- Mediterranean Sea (excluding Alboran Sea, MC)	Among pops/within groups	47	*Φ*sc = 0.01	0.104
- Black Sea	Among populations	530	*Φ*st = 0.33	**<0.001**
3) three groups, PCoA				
- Northernmost Atlantic (PS + AZ)	Among groups	2	*Φ*ct = 0.36	**<0.001**
- Mediterranean Sea (including Alboran Sea, MC) + Canary Island	Among pops/within groups	47	*Φ*sc = 0.02	0.047
- Black Sea	Among populations	530	*Φ*st = 0.37	**<0.001**
4) five groups, differences within Med				
- Northernmost Atlantic (PS + AZ)				
- western Mediterranean Sea (including Alboran Sea, MC) + Canary Islands	Among groups	4	*Φ*ct = 0.21	**<0.001**
- eastern Mediterranean Sea	Among pops/within groups	45	*Φ*sc = 0.003	0.387
- Adriatic Sea	Among populations	530	*Φ*st = 0.22	**<0.001**
- Black Sea				

Degrees of freedom (df), F-statistics and P-values are reported. Significant P values are shown in bold. Population abbreviations correspond to those reported in Table 1

The neutrality tests provided evidence of departure from mutation-drift equilibrium, since all the tests (Tajima’s D, Fu’s F and R2 tests) recorded significant values for the overall population and the three geographic groups (i.e. Atlantic Ocean, Mediterranean Sea and Black Sea: Table 2). Considering separately each population, 27 out of 51 of them (16 by the Fu’s test, only 2 by the Tajima’s D test and 21 by the R2 test) seem to have experienced a recent population expansion (Table 2).

**Fig. 4:**
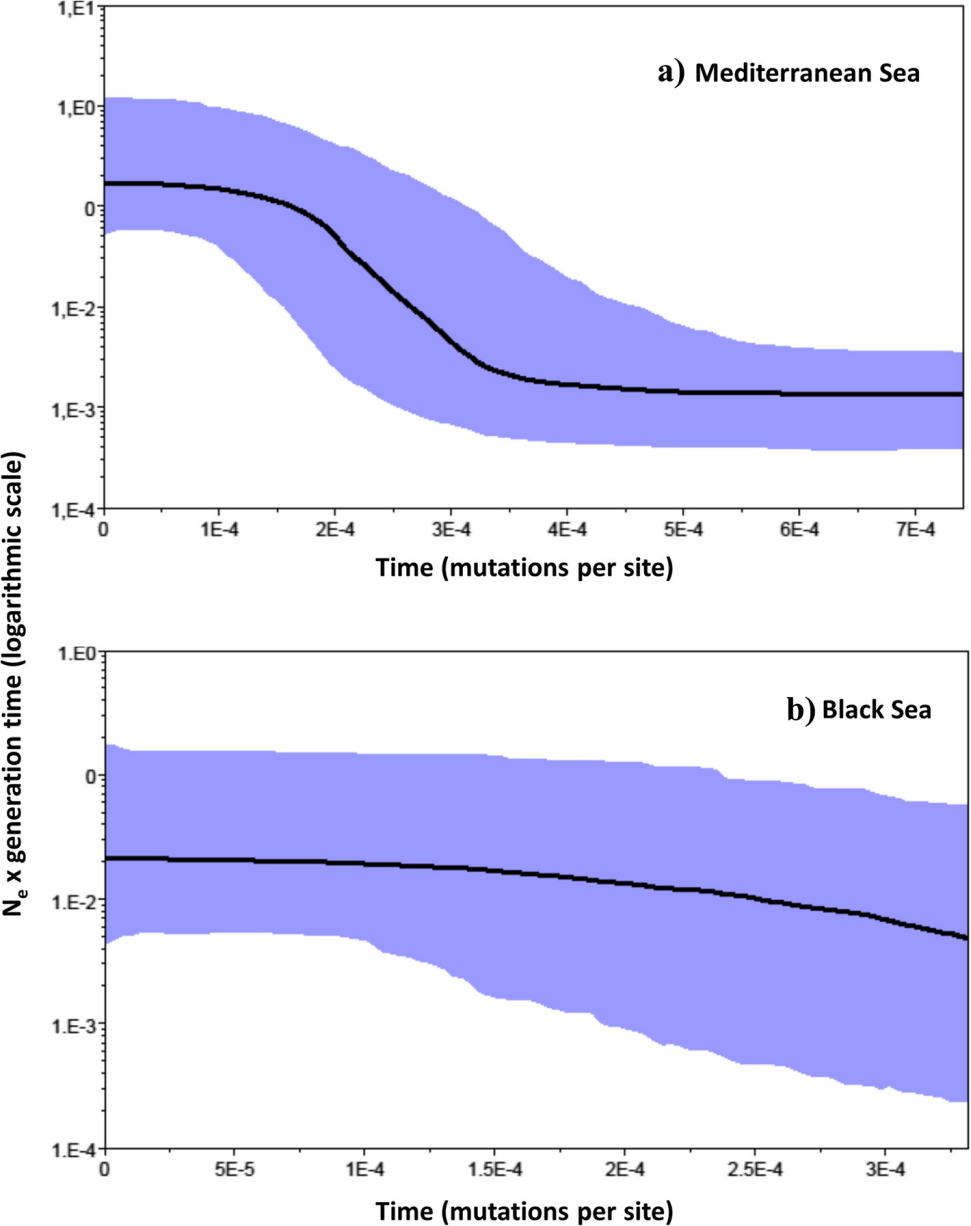
Bayesian Skyline Plots showing changes in effective population size (expressed as effective population size multiplied per generation time) over time (measured in mutations per site) for the Mediterranean Sea (**a**) and Black Sea (**b**) metapopulations. The thick solid line depicts the median estimate and the shaded area represents the highest 95 % posterior density intervals

The population demographic history of the Mediterranean and Black seas was reconstructed also applying the BSP analysis. The Mediterranean metapopulation of *P. marmoratus* showed an increase in population size over time (Fig. 4a), whereas the Black Sea metapopulation showed evidence of demographic stability as explained in Grant [51] (Figs. 4b). Converting mutations units in estimates of years using the CoxI mutation rate of 1.66 % per million years [48], the expansion time for Mediterranean group occurred approximately at about 100,000 years ago (CI interval: 60,000-180,000 years ago).

## Discussion

Most marine invertebrates have a planktonic development and consequently a high potential of long ranging gene flow that may blur historic population structuring (for example see [52]). Among the species with a high potential for gene flow there are also decapod Crustacea of the Mediterranean Sea and the north-eastern Atlantic. For example, shallow subtidal crabs of the genus *Xantho* [53, 54] and *Pilumnus* [55], the hermit crabs *Pagurus excavatus* and *P. alatus* [56], and the pelagic shrimps *Parapeneus longirostris* and *Plesionika heterocarpus* [56] show little or no geographic structure within their distribution range. *Pachygrapsus marmoratu*s is another example of species for which genetic homogeneity or only weak structure had been recorded until now, when analysed with mtDNA across a meso-scale geographic area [20, 21]. However, since these previous studies included few populations covering only parts of the species’ distribution range, the question arose, if a larger dataset with several hundred individuals and covering most of the species’ distribution range, would shed further light on its population genetic structure and unveil phylogeographic patterns.

This was realized in a joined effort in the present paper by investigating phylogeographic and population genetic patterns within the marbled crab *P. marmoratus* with more than 550 individuals from 51 populations distributed from the Atlantic Ocean to the Black Sea. Our results clearly reveal genetic differentiation of populations from the Black Sea from those of the Mediterranean Sea and Atlantic Ocean. The distribution of genetic variation in *P. marmoratus* is thus strongly determined by the biogeographic barrier between the Aegean and Black seas, known to restrict dispersal for many marine species at past and present times (reviewed in [3]).

We also recorded the occurrence of a diverging Atlantic lineage comprised of the two more northern Atlantic populations analysed in this study (i.e. the Portuguese populations from Sesimbra and Azores Islands). Interestingly, the here included population from Canary Islands, which is situated southwest to the Gibraltar Strait, does not appear to belong to the Portuguese Atlantic lineage. A similar latitudinal phylogeographic break has been reported for two limpets of the genus *Patella* [57] and it was explained by the lack of suitable habitat between Iberian and Atlantic African shores for rocky shores animals. Notwithstanding the notable geographic distance, the Canary Island population results to be closely related to the Alboran and Balearic populations and genetically not distinguishable from the Mediterranean cluster. This could be a historical signature, due to the retention of ancestral haplotypes in the Atlantic and Mediterranean lineages as well as incomplete lineage sorting, masking the genetic separation of the Mediterranean populations from this Atlantic population. Nevertheless, we cannot exclude that the Mediterranean-Atlantic transition may currently act as a barrier for *P. marmoratus* larvae, as reported for other marine species (reviewed in [3]). But a certain permeability may maintain a level of population admixture high enough to allow connectivity between African (including nearby Canary Islands) and Mediterranean populations. In any case, our population from the Alboran Sea is genetically homogenous with respect to the other Mediterranean populations and this result indicates that a potential Mediterranean-Atlantic transition would be located at the Strait of Gibraltar, and not at the Almería-Oran front as reported for many other species (see [3]). Sampling at finer geographic scale along the north-western African coastline, the Atlantic Portuguese and Spanish shores, the Alboran Sea and the Gibraltar Strait will be needed to define both the geographic distribution of Atlantic lineages and the location of the Mediterranean-Atlantic transition. In any case, our data show for the first time the occurrence of a separate lineage within the Atlantic Ocean, corresponding to our sampling sites of the Portuguese mainland and Azores Island. It is interesting that the peripheral Atlantic population of the Azores cannot be statistically distinguished from the continental population from Portugal. Due to the marginal and isolated position of the Azores (approximately 1500 km from mainland Portugal), this result is rather unexpected. Many genetic studies on fishes reported strong genetic segregations of Azores’ population from continental ones [58]. However, we cannot disregard that only two haplotypes, one of which a singleton, were sampled in individuals from this archipelago. This could be due to a founder effect and considered as a first signal of an isolation process.

In this study, we report for the first time sharp geographic breaks in the genetic composition of *P. marmoratus* individuals. Previous population genetic studies based on the same mtDNA marker recorded no or only a subtle separation between Mediterranean and Atlantic populations [20, 21]. These former studies lacked large numbers of samples from the eastern Mediterranean and Black Sea. This indicates that phylogeographic studies based on high numbers of populations/individuals covering an extensive geographic area allow both to reveal genetic structure that otherwise may remain hidden and to exhaustively examine which evolutionary and demographic forces have shaped the observed patterns of genetic diversity across species’ ranges. Overall, our genetic diversity and historical demographic results suggest that the population genetic structure of *P. marmoratus* is the result of the complex geological history of Mediterranean Sea and adjacent seas, past isolation due to Pleistocene glaciations, reduced current gene flow in association with the main geographic boundaries, and incomplete lineage sorting.

As clearly shown by the network and genetic diversity indices, Mediterranean populations present higher average levels of intraspecific genetic variation in comparison to Atlantic and Black Sea populations. This result could be partially produced by the overall larger Mediterranean sample size. However, possible differences in evolutionary history in the different basins may argue against the possibility that it is a simple sampling artefact. The low haplotype and nucleotide diversities observed in the Black Sea and Atlantic population can also be the likely result of a strong bottleneck and founder effect, due to past and current isolation.

Following the opening of the Gibraltar Strait, 5.33 million years ago, most of the Mediterranean marine fauna and flora arrived from the Atlantic Ocean [59]. It is currently unknown if *P. marmoratus* followed this route and expanded within the entire Mediterranean Sea or if it survived the Messinian Salinity Crisis in local refugia within the Mediterranean Sea, as suggested for *Carcinus aestuarii* by Marino et al. [60], and subsequently expanded to the Atlantic Ocean and Black Sea. In any case, we can suppose that the phylogeographic pattern of *P. marmoratus* has been affected in more recent times by the drastic paleoclimatic events occurring during the Quaternary period. In correspondence to the repeated glaciations and the associated sea level changes during this time, the contact between the Mediterranean Sea and adjoining epeiric seas was likely interrupted, while during interglacial periods it was plausibly re-established. Based on the geographic distribution of haplotypes, we suppose that the main direction of these fluxes was from the Mediterranean to the Atlantic Ocean and Black Sea, as two of the most common haplotypes of the Mediterranean Sea are represented in low percentages in Atlantic and Black Sea populations (i.e. haplotypes 4 and 2, respectively). The BSP results also support the effect of paleoclimatic events on *P. marmoratus* genetic composition, since a clear sign of demographic expansion was found for the Mediterranean metapopulation, dated around 60,000-180,000 years ago, i.e. long before the Last Glacial Maximum. In contrast, the Black Sea lineage showed evidence of demographic stability according to the BSP. The Black Sea was a freshwater lake during the Last Glacial Maximum [12] and thus inhospitable for true marine species. Hypothesising that the present colonization of the Black Sea occurred after its last connection with the Aegean Sea, i.e. within the last 10,000 years, our demographic analysis suggests that this process was not accompanied by a sudden population expansion.

Our study recorded genetic homogeneity of *P. marmoratus* within the Mediterranean Sea, as already reported from previous studies based on the same mtDNA marker [20, 21]. Therefore, any geographic transitions present at the boundaries among Mediterranean basins and sub-basins do not seem to act as genetic barriers for *P. marmoratus.* Larvae of *P. marmoratus* spend approximately one month in the water column and appear to be able to maintain connectivity among populations, likely following a stepping-stone model, i.e. the most common model for marine species with wide distribution ranges [61, 62]. However, this result is anything but foreseen. In fact, many studies detected the effects of intra-Mediterranean transitions, especially at the Strait of Sicily between western and eastern Mediterranean, on pelagic and benthic species’ population genetic structures (reviewed in [3, 60, 63]). This confirms that in the marine realm dispersal and connectivity are complex phenomena that cannot be generalised for a certain geographic area, since they strongly depend on the life-history and biological traits of the studied species (see [64]).

High level of gene flow can be the reason for lack of substructure within the Mediterranean Sea. However, we have to keep in mind that genetic studies based on microsatellite polymorphisms [21–24] reported genetic heterogeneity among populations of *P. marmoratus* at local scale, even if without clear association to geography. Fratini et al. [23] hypothesised that larval retention and sweepstake effect could be plausible reasons of limited genetic exchange among the islands forming the Tuscan Archipelago (Tyrrhenian Sea). The finding of local-scale genetic differences in microsatellites could indicate that mtDNA studies may underestimate *P. marmoratus* population structure. Notwithstanding, mtDNA markers do not produce *a priori* higher estimates of gene flow than hypervariable nuclear markers, as recently stated in Karl et al. [65]. Further population genetic studies based on microsatellite polymorphism and including populations from all the Mediterranean sub-basins could help in clarifying the level of present day gene flow across the entire Mediterranean Sea.

## Conclusions

This is the first study to provide clear evidence of genetic differentiation of *Pachygrapsus marmoratus* across its distribution range and unravel the existence of three distinct phylogeographic lineages, corresponding to the Portuguese Atlantic Ocean, the Mediterranean Sea plus Canary Islands, and the Black Sea. Their genetic distinctiveness is likely the consequence of geological and paleoclimatic processes, historically affecting Mediterranean Sea and adjacent waters, and it may currently be maintained by present-day geographic breaks and species-specific biological traits. Local adaptations to climatic conditions and environmental parameters may contribute to increase the recorded genetic differences among lineages. Morphometric and eco-physiological studies on individuals from the three geographic clusters should be interesting for corroborating this hypothesis.

## Additional files

Additional file 1: Variable sites among 74 mitochondrial haplotypes of *P. marmoratus* from Atlantic Ocean, Mediterranean and Black seas (*n* = 587). All haplotypes are compared with haplotype 1. *, indicates identical nucleotides; PS, parsimonious sites; aa1 and aa2, aminoacids in haplotype 1 and after mutation. Haplotypes 1-33 and 34-49 correspond to those reported in Fratini et al. [20] and Deli et al. [21], respectively. (XLSX 24 kb) Additional file 2: Pairwise *Ф*st values based on Tajima and Nei distance model. In bold are indicated significant values after 10,000 corrections. (XLSX 30 kb) Additional file 3: Dataset supporting the conclusions of this article (text file formatted as Arlequin input). Haplotypes 1-33 and 34-49 correspond to those reported in Fratini et al. [20] and Deli et al. [21], respectively. (ARP 52 kb)
